# Negotiating language in family texts: case-study of transnational families in Finland

**DOI:** 10.3389/fpsyg.2025.1672423

**Published:** 2026-01-08

**Authors:** Gali Bloch

**Affiliations:** 1 Department of Languages, University of Helsinki, Helsinki, Finland; 2 David Yellin Academic College of Education, Jerusalem, Israel

**Keywords:** FLP, Nordic countries, Modern Hebrew, translanguaging, multilingual familylect

## Abstract

Transnational families face complex language dynamics, particularly when adapting to new linguistic environments. This study examines text messaging practices of six bilingual Russian-Hebrew-speaking parents and their multilingual children living in Finland. Through micro-interactional approach, it investigates how digital translingual practices function as a tool in constructing unique familylects. The study categorizes translanguaging practices by form—isolated insertions, sentence-level switches, and complex multilingual constructs—and by function—local/temporal, phatic, and address terms—which serve to enhance the clarity of practical information, reinforce familial bonds, and reflect multicultural identity. This research contributes to the field of multilingual digital communication by illustrating how messaging language choices serve as contextualization cues, enhancing the overall meaning and emotional resonance of conversations. The findings reveal how transnational families adapt their communicative practices to maintain connections and cultural identity, illustrating the role of text messaging as a locus for linguistic and social negotiation.

## 1 Introduction

Family language dynamics is unique and complex within each family, especially in transnational multilingual families, where diverse linguistic practices shape family identities and relationships. These language practices evolve in everyday interactions, and multilingual families often develop a distinctive linguistic repertoire—a familylect (Van Mensel, 2018). Familylects are shaped by integration of various languages and cultural resources, allowing families to express meaning and identity in dynamic ways. In this view, families are not merely physical settings but social spaces where multilingualism plays a central role in defining relationships and communication (Lanza, 2021). This ongoing negotiation is guided by Spolsky’s (2004) language policy model which outlines language ideologies, management, and practices at macro, meso, and micro levels, providing a key framework for understanding Family Language Policy (FLP). As was and remains the basic model, Spolsky (2021) defines language practices as ‘the choices of language variety made by speakers in a community’ (Spolsky, 2021, p. 21), shaped by both internal dynamics and external societal influences (Spolsky, 2004, 2021). From the sociology of language perspective, Boutet et al. (2025) argue that language practices are socially organized phenomena that cannot be understood as purely individual acts but need to be situated in their historical and social contexts, shaped by relations of domination and subordination.

Within families, these broader perspectives intersect with specific strategies for managing multilingualism. Traditionally, many multilingual families have adhered to several strategies, among them OPOL and ml@h, with each parent using their heritage language^1^ exclusively, thus treating bilingualism as double monolingualism (Heller, 2007). In contrast, translanguaging offers a more flexible and natural approach, allowing speakers to draw on their full linguistic repertoire rather than follow strict language boundaries (García and Baetens Beardsmore, 2009). Recent studies have shown a shift from strict language separation to the incorporation of translanguaging, illustrating how multilingual families adapt their language practices to reflect child agency and cultural identity. Soler and Zabrodskaja’s (2017) analysis of empirical data from Spanish-Estonian-speaking families in Tallinn illustrates how children’s agency, already evident as young as kindergarten age, weighed heavily in determining languages to be used in settings where the children had greater scope to influence parental choices, such as particular games and bedtime stories. Nenonen (2024) also illustrates how child agency shaped family language practices: in a family with a Russian-born mother, an Italian-born father, and their Finnish-born 8-year-old son attending the Jewish school of Helsinki, the child’s persistence and rejection of alternatives, such as parents switching to English, reinforced the OPOL strategy and maintained language boundaries within the family. Istanbullu’s (2024) study of phone conversations across three generations—in which the first and second generations were born in Turkey, with Arabic being the grandparents’ and the parents’ language of first socialization, and the grandchildren were born in Europe—reveals that grandparents often aligned to the language choices of their grandchildren, adopting French or German instead of Arabic or Turkish. Conversely, the grandchildren were experimenting with innovative, unexpected forms of Arabic and Turkish, demonstrating translanguaging instances shaped by child agency.

In this context, mobile phone communication, especially through text messaging, has become a vital tool for multilingual families (Pekarek Doehler, 2011). Text messaging serves as an important space for multilingual expression, where families can easily switch between languages and scripts, reflecting the adaptive nature of their communication, thus providing a dynamic environment for the development of familylects (Morel and Pekarek Doehler, 2013). Recent large-scale evidence from Finland shows that while texting in the two national, high-status languages, Swedish and Finnish, is the most common family app practice, children sometimes refrain from texting in other languages used in the family if these languages have lower status, limited educational support, or different writing systems (Palviainen and Räisä, 2023).

This qualitative case study examines text message communication in transnational Russian–Hebrew-speaking families residing in Finland. The data collected between October and November 2024 consist of WhatsApp communication excerpts provided by six participants, focusing on translanguaging practices. The research questions that guide this study are: How do transnational multilingual families shape and negotiate everyday language practices in multilingual text messaging communication? What are the primary linguistic forms of translanguaging observed in their WhatsApp communication? What communicative functions do these translanguaging practices serve? By examining how transnational multilingual families use text messaging in WhatsApp communication, this article explores how the translanguaging practices of the family members contribute to the formation of a multilingual familylect. Through this lens, the study highlights the role of WhatsApp communication in shaping family identities, cultural belonging, and emotional connections in multilingual families (Piller and Gerber, 2021).

This article is organized as follows. The theoretical background Section 2 explores translanguaging practices in multilingual families and multilingual familylects; it also provides an overview of Israelis and Hebrew, with a focus on Generation 1.5 speakers. Section 3 presents the data, participant details, and explains the methodology. Section 4 presents the findings on the forms and functions of translanguaging observed in the data. The final section discusses the key findings, summarized in the conclusion.

## 2 Theoretical dimensions

Mainstream parenting literature often promotes language separation when raising multilingual children, with various strategies tailored to different family contexts (Piller and Gerber, 2021). A common approach is ‘one parent–one language’ (OPOL), where each parent speaks either their heritage language or the societal language (Reichmuth, 2024). Other strategies include ‘Minority Language at Home’ (ml@h), with the heritage language used exclusively at home and the societal language outside, as well as ‘one parent–two languages’ (OP2L), and additional approaches (Ballinger et al., 2022; Ruiz Martín, 2017). Rooted in monolingual ideologies, these approaches, popular and often supported by extended families, view multilingualism as multiple monolingualism separating languages (Heller, 2007). However, scholars have challenged this perspective, arguing that multilinguals are not simply two or more monolinguals in one, and in practice, separating languages and providing distinct support across family, societal, and educational contexts is difficult (Reichmuth, 2024; Protassova, 2018).

In contrast, translanguaging reflects the fluid and dynamic use of a speaker’s entire linguistic repertoire, without enforcing or adhering to rigid rules about using a specific language exclusively. Recent studies have highlighted the adaptability of multilingual families, showing a shift from strict practices to incorporating translanguaging, shaped by such factors as child agency and cultural identity (e.g., Karpava et al., 2025; Nenonen, 2024). This evolution underscores the importance of recognizing and embracing the complex, integrative language practices that more accurately represent multilingual communication.

### 2.1 Translanguaging

Language in the 21st century can no longer be viewed as a fixed code, but as the flexible use of all available semiotic resources by individuals who must act as active meaning-makers and communicators in a digitally connected world (García and Wei, 2018). García and Baetens Beardsmore (2009) define translanguaging as dynamic use of a bilingual or multilingual speaker’s full linguistic repertoire, characterized by fluid communication across languages in everyday interactions. It shifts focus from languages as distinct systems to the observable practices of multilingual individuals and communities, centering on the speaker as an active meaning-maker who selects elements from their dynamic linguistic repertoire to communicate and interpret experiences (García and Wei, 2018). In many, if not most, contexts worldwide, translanguaging is a natural and commonplace aspect of interactions within the same multilingual culture. Translanguaging involves using one’s unique linguistic repertoire freely, without being constrained by socially or politically defined language names or labels. Language labeling is frequently arbitrary, therefore the translanguaging perspective views the question of which language is being used as insignificant, emphasizing the blending of linguistic and non-linguistic resources in communication (Wei, 2018).

Treffers-Daller (2024) highlights the urgent need for further investigation into translanguaging, particularly in three key areas: its theoretical conceptualization, its practical applications in research and education, and the development of clear diagnostic criteria for identifying translanguaging practices. Empirical research further supports the idea that translanguaging is a dynamic and strategical approach within multilingual families. A recent study on Russian-speaking multilingual families in Cyprus, Estonia, and Sweden highlights translanguaging as a dynamic strategy that bridges linguistic gaps, enhances adaptability, accommodates diverse sociolinguistic contexts, and fosters both emotional well-being and linguistic identity of all family members (Karpava et al., 2025). In Soler and Zabrodskaja’s (2017) study of a Spanish-Estonian family, the parents, despite idealizing the OPOL strategy, engaged in significant translanguaging, particularly within couple interactions, as their relationship had initially developed in English, with this practice further amplified by the presence of children. The OPOL policy facilitated the inclusion and simultaneous use of all linguistic resources within the family context. Similarly, a recent case-study of a Russian-, Italian-, Hebrew-, English, and Finnish-speaking family in Finland showcases a shift from a strict OPOL approach to gradually incorporating translanguaging, reflecting the family’s adaptability and the dynamic nature of language practices shaped by child agency and cultural identity (Nenonen, 2024).

#### 2.1.1 Trans-scripting of translanguaging in digital communication

In digital spaces, translanguaging involves trans-scripting, representing one language’s features in another’s spelling or script; this practice enables its users to exploit their full literacy repertoires and generate new forms of communication (Androutsopoulos, 2009). Alphabets function as writing systems to represent speech sounds, but their role extends beyond simple transcription, encompassing socio-cultural dimensions, such as symbolic significance (Kress, 2003) or political implications (Baron, 2002). For instance, Wei (2018) examines computer-mediated communication (CMC) in New Chinglish, where Chinese speakers of English repurpose common English expressions with entirely new meanings to suit their communicative needs, while incorporating both scripts. The corpus of New Chinglish features creative word formations and expressions that, while generally adhering to English morphological structures, are infused with uniquely Chinese interpretations and cultural nuances. Androutsopoulos (2009) investigates the online usage of Greeklish, the Romanized form of Greek, highlighting phonetic and orthographic transliteration patterns, its association with technological competence and cosmopolitan identity, and ideological debates framing it as either a threat to or enrichment of Greek language and identity. Spilioti (2022) examines reverse trans-scripting in CMC, focusing on how users create stylized speech and social personas through hellenized English—Engreek. The findings suggest that trans-scripting enables users to manipulate language creatively, reflecting their social identities and aligning with broader discussions on language diversity in the digital age. Elhija (2023) explores trans-scripting digital communication practices between Arabic and Hebrew, finding such practices most common in the fields of education, employment, and politics. Trans-scripting instances include both toggling the two keyboards and trans-scribing one language using the script of the other, characterized by adaptations of Hebrew to Arabic phonology, but not vice versa. The study findings also highlight the role of context and intimacy in language and script choice.

### 2.2 Multilingual familylect

Families as social structures can be understood as ‘constructing and inhabiting dynamic systems of meaning-making’ (Purkarthofer, 2019, p. 725), where members bring together their lived language experiences into shared practices that evolve over time and across contexts (Purkarthofer, 2019). Gordon’s (2009) assertion that ‘it is not possible to fully comprehend the private language of any family other than our own’ (p. 76) underscores the deeply personal and contextually embedded nature of familylects. The term familylect, originally introduced by Søndergaard (1991), refers to the unique linguistic patterns within a family, where members consciously explore linguistic boundaries. Gordon’s (2009) definition of familylect emphasizes the unique linguistic repertoire shared within a family, rooted in collective memory and prior language experiences. It constructs family identity and boundaries through repeated, family-specific language practices, highlighting that families are culturally created through discourse rather than biologically predetermined. In multilingual families, the shared practices that shape a collective family culture may incorporate elements from multiple languages, creating a multilingual familylect. The combination of family-specific language practices with translingual practices results in a dynamic and flexible communication system that reflects both the family’s uniqueness and multicultural identity. As defined by Van Mensel (2018), a multilingual familylect, or multilingual family repertoire, is the unique linguistic repertoire of a multilingual family, characterized by communicating across languages, language choice patterns, and shared linguistic features. It is shaped by a range of practices, some of which are shared with monolingual families, such as language rituals, adopting each other’s voices, and establishing family-specific routines or traditions. However, multilingual families also engage in practices unique to their context, such as situation-specific language use, creating new language forms by combining languages, and children challenging the language choices made by adults (Van Mensel, 2018).

### 2.3 Israelis and Hebrew

Modern Hebrew is the predominant language in Israel, serving as the first language for 49% of the population, and as the common language of communication for 92% of Israelis (Central Bureau of Statistics, 2022). Israelis residing abroad typically continue speaking Hebrew in their homes and with friends, and often maintain connections to Israeli culture and identity. However, a gradual shift away from Hebrew is evident, influenced by a variety of factors including the size of the diaspora, higher prestige of other languages, level of professional proficiency in the diaspora, changes in Jewish identity, and lack of clear objectives in Hebrew instruction (Spolsky, 2016; Nevo and Verbov, 2011). An additional important yet understudied factor in the shift away from Hebrew is migrant parents’ bilingualism, which, in a third-language environment, enables them to prioritize between two heritage languages for home maintenance. In a case study of three generations of Russian-speaking Israelis, the second-generation bilingual Hebrew-Russian parent who migrated to Portugal maintained both heritage languages with the child; however, the child’s Russian proficiency was described as ‘very good’, while Hebrew was assessed as ‘decent’, possibly reflecting parental language priorities abroad (Protassova and Yelenevskaya, 2024). In a recent study on Israelis with a Post-Soviet State (PSS) background in Finland, parental bilingualism significantly influenced family language dynamics, highlighting a shift away from Hebrew as many parents prioritized Russian over Hebrew in heritage language maintenance (Bloch, 2024b).

Indeed, Russian, spoken by approximately 15 to 20% of the population of Israel, shapes the language dynamics within families with an PSS background both in Israel and abroad (Protassova and Yelenevskaya, 2024). Starting with the collapse of the USSR in 1991 and till 2000, around a million people immigrated to Israel from PSS (Perelmutter, 2018). Most children of this wave, known as Generation 1.5, became ‘skewed bilinguals’ within their first decade in Israel, primarily using Russian only with Russian monolinguals, while progressively shifting to Hebrew with younger family members, friends, and their own children (Remennick and Prashizky, 2019). For communication amongst themselves, they developed a code-mixed language—Israeli Russian—that uses Russian as the matrix language, integrating Hebrew elements through code-mixing and adaptations in phonology, morphology, and syntax (Naiditch, 2008). Often viewed as a ‘Low’ variety in contrast to Modern Standard Russian’s ‘High’ status, Israeli Russian serves everyday communication functions. Despite criticism from speakers when discussed with outsiders, it plays a crucial role in strengthening community bonds (Perelmutter, 2018). While there is extensive research on Israeli Russian in Israel, studies of Russian-speaking Israelis’ language practices in third-country contexts remain scarce (Rebhun, 2016; Remennick, 2019).

Determining the exact number of Israelis living abroad is highly challenging since there is a lack of direct data on the annual number of Israelis explicitly intending to emigrate. However, it is estimated that ~10% of Israel’s population resides outside the country at any given moment, with upward trend since late 2023 (DellaPergola and Lustick, 2011; DellaPergola, 2025). Data on the languages spoken by these emigrants is elusive, or perhaps non-existent.

As of December 2024, Finland had 990 Israeli citizens (with 569 holding dual citizenship). Out of them, 536 self-identified as Hebrew speakers, and 228—as Russian speakers (Official Statistics Finland, 2024). Finland’s language registration system, which records only one mother tongue, complicates the accurate count of bi- and multilinguals (Palviainen and Bergroth, 2018). While most of the Israelis born in PSS registered their mother tongue as Russian, 27 reported it as Hebrew (Official Statistics Finland, 2024). In a recent survey of Hebrew-speaking parents in Finland (Bloch, 2024a), 10 out of the 36 participants self-identified as Generation 1.5 Russian–Hebrew bilinguals.

## 3 Data and methods

This case study examines the FLPs in transnational Russian–Hebrew speaking families residing in Finland. It adopts a qualitative approach to examine the real-life language practices of the participants and their children.

### 3.1 Participants

The participants were recruited among Hebrew-speaking parents in Finland (c.f. Bloch, 2024a), as well as the community network. Ten participants were selected based on specific criteria: born in the PSS, migrated to Israel between the ages of 6 and 15, considered themselves natively proficient in both Hebrew and Russian, and had school-aged children. Of these, one participant withdrew, and three reported strictly monolingual family messaging (Hebrew or Russian), verified by 3–4 screenshots each (available upon request). Since no translingual practices were observed, they were excluded from the study.

The participants in this study are reported collectively to ensure their anonymity, as individual details, such as exact ages, specific durations of residence in Finland, and some other personal characteristics, were omitted due to the small size of the community and participants’ requests for maximum confidentiality. All six participants in the study (four identified as females and two as males) were aged between 40 and 50 years and had two to three children, ranging from 7 years old to young adults living independently, though only exchanges with children aged 10 and above were examined in this study. As minors, all participants had migrated to Israel between the ages of 7 and 15, classifying them as Generation 1.5. Later, as parents of at least one child, they migrated to Finland, with the duration of their residence in Finland ranging from 1.5 years to over 20 years. Two participants were a married couple; in the four remaining cases, the participants’ spouses were PSS-born native Russian speakers who moved to Israel as young adults (between the ages of 18 and 21) and spoke advanced but non-native Hebrew. All participants reported being bilingual, with native proficiency in both Russian and Hebrew. Their self-reported level of Finnish varied and was not always consistent with the length of their stay in Finland. The participants resided in various locations in Finland, both in main cities and small towns with a population under 10.000.

### 3.2 Data

The six participants shared excerpts from their WhatsApp communications with their children. Each participant submitted between 5 and 18 screenshots of recent WhatsApp conversations, containing excepts they considered interesting instances of usage of more than one language in a conversation, totaling 57 screenshots, with 5–8 messages per screenshot, collected between October and November 2024. Participants provided contextual explanations, both during the submission and later upon request.

**Figure 1:**
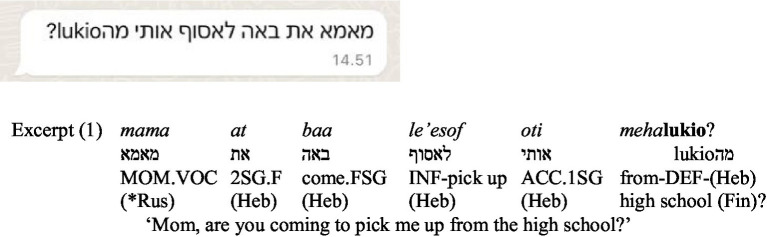
Screenshot of Excerpt (1).

The data were translated into English and transcribed according to Leipzig Glossing conventions (Comrie et al., 2008), with the language(s) of each word indicated in brackets within the glossing. The three scripts employed in family messaging were distinguished as follows: Cyrillic in plain text, Roman (or Latin), representing both Finnish and English, in bold text, and Hebrew in italics (Figure 1; Excerpt 1). Terms attributable to several languages were marked with an asterisk (Istanbullu, 2021).

The micro-interactional approach is used to explore how language choices within transnational multilingual families serve immediate interactional and conversational goals (Smith-Christmas, 2014). This approach emphasizes the local, situational use of linguistic features as contextualization cues that help negotiate meaning within interactions (e.g., Gafaranga, 2012). The micro-interactional approach operates at multiple levels of analysis. On the sociocultural level, it focuses on shared contexts and cultural knowledge (Smith-Christmas, 2014). In written multilingual conversations, speakers rely on their shared linguistic and cultural backgrounds to create meaning and foster engagement. Translanguaging, in this context, is a strategic tool that taps into the interlocutors’ familiarity with multiple languages, making the communication more resonant and meaningful. On the interactional level, the micro-interactional approach focuses on interactional goals, with translanguaging strategically employed to achieve specific communicative objectives, such as clarifying a point or shifting the direction of the conversation (Gafaranga, 2012). Interactional goals are achieved through different forms and functions of translanguaging practices. While on the sociocultural level, instances of translanguaging serve to align with the social and cultural expectations of the participants, on the discourse level, they enhance the efficiency and effectiveness of communication. The micro-interactional approach assesses communication efficiency by analyzing how participants achieve immediate interactional goals through language choices: mutual understanding, manage misunderstandings, and maintain conversational coherence (Abe and Roever, 2020). Together, these levels offer a comprehensive framework for investigating CMC communication within the participants’ families.

In this study, the micro-interactional approach was applied to examine multilingual WhatsApp communication, focusing on how six participants and their children use translingual practices to achieve specific conversational goals, including practical functions, shared cultural and linguistic knowledge, and the emotional and social functions of each language. The recurring conversational goals were identified according to the theoretical frameworks used in examining translingual practices within multilingual transnational environments across various scholarly works and publications.

## 4 Findings

This section examines the different forms and functions of translanguaging practices observed in the data. It explores three main forms of translanguaging: complex multilingual constructs, isolated word or phrase insertions, and sentence-level switches. Following this, the section discusses the functional characteristics of these practices, highlighting their roles in local and temporal expressions, phatic actions, and address terms.

### 4.1 Forms of translanguaging

The recurring forms of translanguaging in the data were categorized into three types. The most frequent type was represented by multiple spontaneous alternations between the languages within one sentence which can be completely understandable only to the family members, thus forming a familylect. The other two types of translanguaging included isolated words or expressions from each of the three or four languages available, and sentence-level switches. In many cases it was difficult to adapt each case of translanguaging to only one category, since they played two roles simultaneously—to convey the meaning in a more practical way, and to underscore the uniqueness of each communication act through translingual practices forming familylects.

#### 4.1.1 Complex multilingual constructs

**Figure 2:**
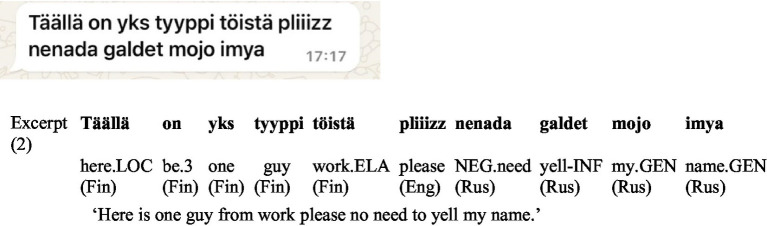
Screenshot of Excerpt (2).

A substantial portion of translingual practices within the families involves the dynamic blending of vocabulary, grammar, and scripts from multiple languages. Trilingual instances were a common feature of WhatsApp communication across families, with the sources of linguistic transfer varying significantly (Excerpt 2, Figure 2).

This translingual instance demonstrates a dynamic interplay of languages: the child’s message written in the Roman script begins with colloquial Finnish (‘*Täällä on yks tyyppi töistä’* [here is one guy from work]), establishing local context. Mid-turn, the child inserts an English isolated sedimented borrowing *pliiizz* to intensify the request in an informal register—a feature particularly associated with youth and SMS communication (Morel and Pekarek Doehler, 2013). The request concludes with a citation of the request itself in Russian ‘*nenada galdet mojo imya’* [no need to yell my name]. Thus, within a single turn, the child orients first to the Finnish work environment, then to global youth style through English, and finally to the intimate family domain through Russian. This layering illustrates a pragmatic distribution of languages across sequential functions (cf. Gafaranga, 2012; Smith-Christmas, 2014). When asked for clarification, the parent could not recall whether the child was citing a third person or referring to themselves. In this instance, the child exclusively used Roman script, but the spelling conventions varied. For the Finnish segment, standard Finnish spelling was applied; however, in the Russian portion, the spelling adhered to English conventions—for example, the letter ‘y’, which represents different sounds in Finnish and English, was employed in the Russian word for ‘name’ in line with English spelling.

**Figure 3:**
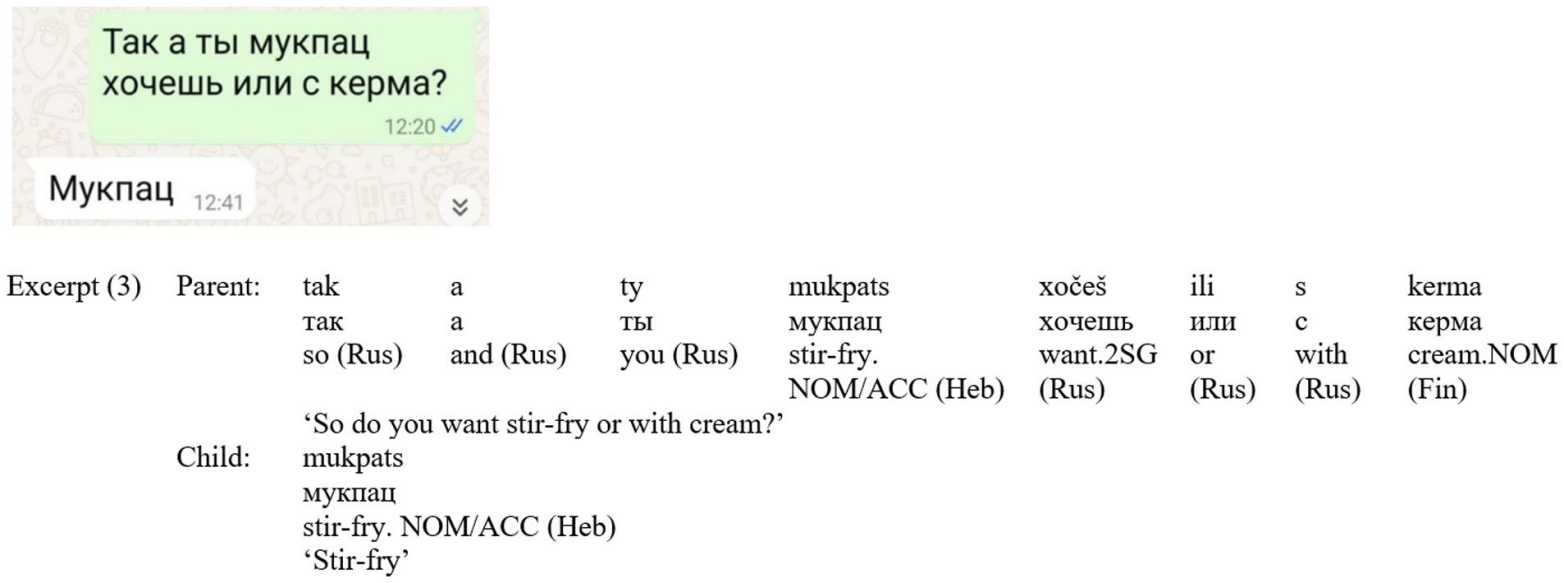
Screenshot of Excerpt (3).

Excerpt (3) (Figure 3) also incorporates three languages, with Russian serving as both the grammatical and script base. The Russian frame hosts Hebrew *mukpats* [stir-fry] and Finnish *kerma* [cream]. The child responds minimally with *mukpats* in Hebrew, confirming the preferred choice. The parent offers multilingual options, and the child confirms with an echo, showing alignment and efficiency; the parent’s trilingual turn reflects shared family knowledge (cf. Lanza, 2021). The use of ‘stir-fry’ in Hebrew can be attributed to a technical constraint— this term is rarely used in Russian, has a lengthy and complex spelling, and is often replaced by the English equivalent. In contrast, the Hebrew term is concise and widely recognized in Israeli Russian context. The inclusion of ‘cream’ in Finnish can be attributed to a familylect, specifically referencing a particular type of Finnish cream commonly used within the family.

Another notable feature of linguistic forms in SMS-mediated communication is mixing graphemic conventions (Morel and Pekarek Doehler, 2013). In Excerpt (1), Hebrew serves as both the grammatical and script base for most of the sentence. However, the address term *mama* [mom] appears in Russian, transcribed using the Hebrew alphabet. The script norm of Modern Hebrew is not followed—rather than using diacritics to indicate the ‘a’ sound in the middle of the word (typically omitted in Modern Hebrew), the child employs the letter *aleph* to mimic Russian graphemic norms. The use of the Russian term for *mom* reflects emotional significance, aligning with Istanbullu’s (2021) observations of emotional address in multilingual families, while its transcription in Hebrew script transforms it into a unique element of the familylect. The child then continues in Hebrew, ‘*at baa le’esof oti meha-’* [are you coming to pick me up from the-], ending with *lukio*, a Finnish term for ‘high school’. Excerpt (1) demonstrates how different parts of the message are anchored in different domains: emotion in Russian, request in Hebrew, and location in Finnish. Furthermore, the child toggles the keyboard to provide the precise location for pickup, attributed to a practical constraint. Such cases are relatively common within multilingual families, as once a language or keyboard switch occurs, it can often prompt additional switches rather than returning to the original language. Trilingual speakers, in particular, may transition to a third language, effectively branching out further with each subsequent switch (Clyne, 2003).

#### 4.1.2 Isolated word or phrase insertions

Participants frequently inserted isolated words or short phrases from one language into conversations conducted predominantly in another. These insertions fulfill contextual needs, often reflecting cultural or local nuances associated with specific socio-linguistic environments. The data on these isolated forms align with prior research, which highlights that CMC users tend to incorporate ‘ready-made’ formulas from other languages into their messages. These borrowings often require no morpho-syntactic adaptation, allowing for seamless integration into ongoing interactions. The recurrence of such forms appears to be a distinctive feature of CMC, underscoring its practical and formulaic nature (Pekarek Doehler, 2011; Ndabihonge, 2023).

**Figure 4:**
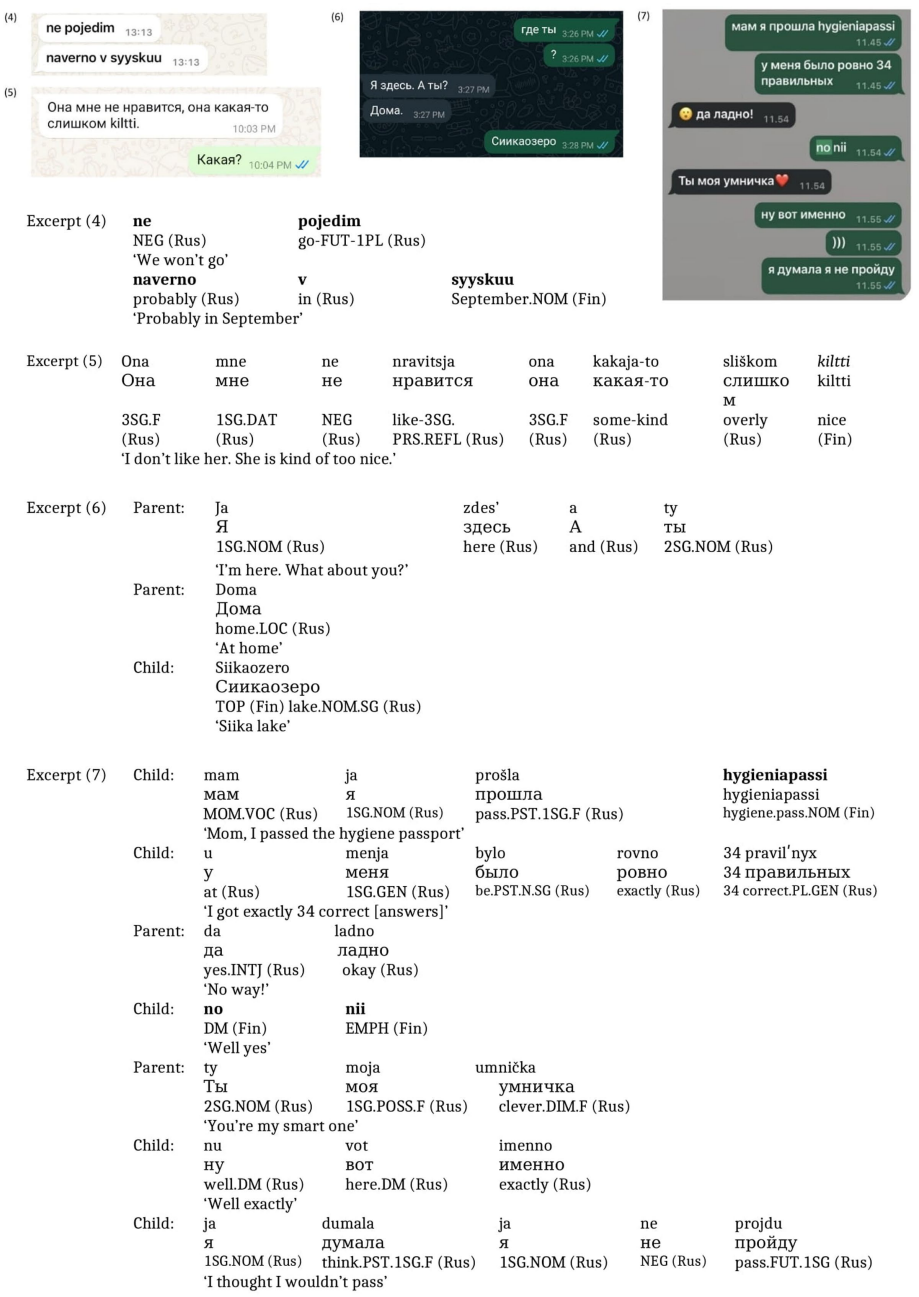
Screenshots of Excerpts (4–7).

In both (4) and (5) (Figure 4), the children engaged in translanguaging, drawing on their language resources asymmetrically (Istanbullu, 2024), primarily relying on Russian while making more limited use of Finnish. In (4), the entire sentence was written in Roman script, with the Finnish word *syyskuu* [September] inserted into a Russian frame, indexing the child’s local and temporal context. In (5), the child toggled between scripts, using Cyrillic for the Russian text and Roman script for the Finnish term. The whole stance is voiced in Russian, but the Finnish adjective *kiltti* [nice/kind] is used deliberately because a Russian equivalent is not readily available and the Finnish term captures precisely the intended nuance. In excerpt (6) (Figure 4), the conversation was conducted entirely in Russian, but a grammatical construction involving a Finnish proper name was used. The child asks the parent in Russian about their location. The parent also uses Russian for a location confirmation (*‘Ja zdes’. A ty? Doma.*’ [I’m here. And you? At home]), while the child responds with a place name *Siikaozero. Siika* refers to the name of a lake, and in Finnish, lake names are written as one word, with ‘lake’ following the proper name. In contrast, in Russian, lake names are written separately. The sequential move shows place-indexing: the parent asks in Russian; the child responds in a mixed form reflecting their Russian framing and Finnish schooling. In excerpt (7) (Figure 4), the child announces success on the hygiene exam in Russian grammar and Cyrillic script, but with the Finnish term for ‘hygiene passport’—*hygieniapassi*. The parent reacts in Russian ‘*da ladno’* [no way], showing surprise, then again in Russian—‘*ty moja umnička’* [you are my smart one]. Interactionally, the child uses Finnish for an institutionally bound term, while the parent responds affectively in Russian. This adjacency pair illustrates how translanguaging distributes pragmatic roles: Finnish for institutional terminology, Russian for emotional evaluation. Transition to Finnish with ‘well yes’ (*no nii*) suggests an alignment with Finnish conversational norms, emphasizing agreement or confirmation.

#### 4.1.3 Sentence-level switches

Translanguaging frequently occurred at the sentence level, with participants alternating between languages to address diverse communicative needs, such as emphasizing a message or adapting to the evolving context within the same conversational thread.

**Figure 5:**
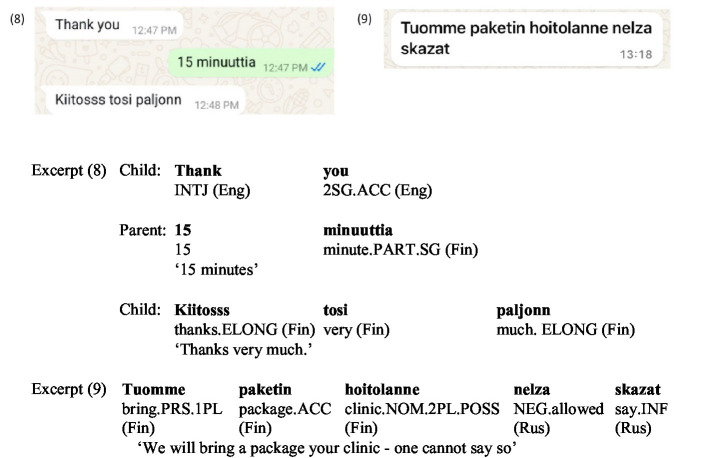
Screenshots of Excerpts (8–9).

In Excerpt (8) (Figure 5), the sequence shows turn-by-turn language adaptation: when the parent approves the child’s request for extra screen time via Family Link, the child first expresses gratitude in English; when the parent specifies the time in Finnish—‘*15 minuutia’*, [15 min], the child aligns by thanking again in Finnish and amplifies their thanks phatically. In excerpt (9) (Figure 5), the child repeats the parent’s Finnish sentence, retaining its grammatical error—nominative instead of illative (‘*Tuomme paketin hoitolanne’*, literally [We will bring the package your clinic]), and then adds a metacommentary in Russian (‘*nelza skazat’* [one cannot say that]), while retaining the Roman script. From the micro-interactional perspective, this example highlights how participants use language as a contextualization cue to negotiate meaning and manage interactional goals in real-time. This intra-sentential change of languages reflects the child’s strategic use of linguistic resources to address contextual demands effectively, with Finnish fulfilling transactional needs and Russian adding cultural or explanatory depth. The use of the Roman script for Russian may demonstrate an adaptation to the digital context, where script choice aligns with communicative convenience and digital norms. On the other hand, it may simply be a matter of convenience, stemming from an unwillingness to toggle between the keyboards.

### 4.2 Functional characteristics of translingual practices

The varied forms of translanguaging illustrate the creative use of linguistic resources observed in the WhatsApp communication in the participants’ families. These practices serve to convey meaning effectively while also emphasizing the uniqueness of each interaction, shaping the distinctive familylects observed in the data. By combining vocabulary, grammar, and scripts from multiple languages, participants address contextual demands and reinforce shared cultural and emotional bonds. Building on this, Section 4.2 examines the functional characteristics of translanguaging, which emerges not as a random act but as a deliberate practice, enabling participants to negotiate meaning across languages, scripts, and cultural contexts. Drawing on Pekarek Doehler’s (2011) research findings, this study highlights three primary functions of translanguaging in multilingual communication: marking of local and temporal expressions; phatic actions, such as greetings or expressions of gratitude; and address terms. While the data revealed a broader range of functions, these three emerged consistently across interactions from all participants.

#### 4.2.1 Local and temporal function

Translanguaging in mobile-mediated communication often occurs in relation to local and temporal expressions, including topographical references and adverbial markers of place and time. This function is especially prominent in texting, where lack of physical presence and asynchronous nature of interactions encourage the use of linguistic resources to convey and share diverse lived experiences (Morel and Pekarek Doehler, 2013). In several instances, participants’ translingual practices can be attributed to its referential function, particularly in its ability to enhance the clarity and precision of meaning conveyed (Lüdi and Py, 2002).

**Figure 6:**
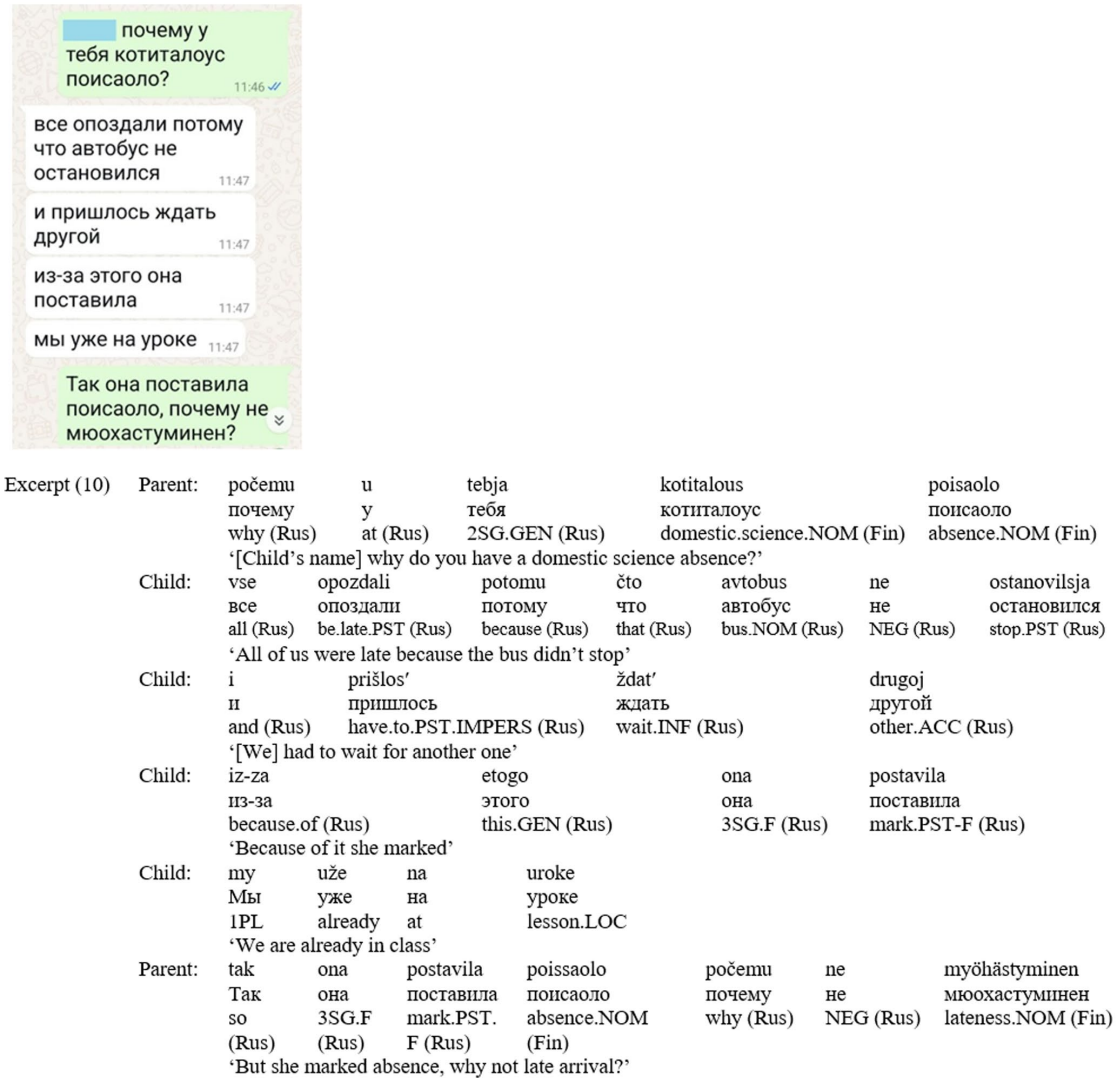
Screenshot of Excerpt (10).

Revisiting Excerpt (1), *lukio* refers to a more specific concept than its Hebrew equivalent *tichon* (high school) within the same communicative context, as it reflects the cultural and educational nuances of the Finnish system. Similarly, in (10) (Figure 6), the dialogue unfolds in the framing of Russian, with three terms from the Finnish educational context used by the parent. The parent uses Russian for formulating the question – ‘*počemu u tebja’* [why do you have], and continues with Finnish terms. *Kotitalous* [home economics], [domestic science] is a Finnish-specific subject that is not part of the Israeli curricula, and differs significantly from its Russian counterpart, ‘labor classes’. Moreover, the term *kotitalous* simultaneously serves as a reference to a specific place (the school) and a time frame shared and understood by both participants. The terms for ‘absence’—*poissaolo*—is also used in Finnish, the way it appears in the school system. The child replies with a multi-turn explanation in Russian; in the last utterance, the parent clarifies the question, again in Russian, and again using two school terms in Finnish – *poissaolo* [absence] and *myöhästyminen* [late arrival], illustrating how Finnish provides terminology while Russian manages the narrative flow.

**Figure 7:**
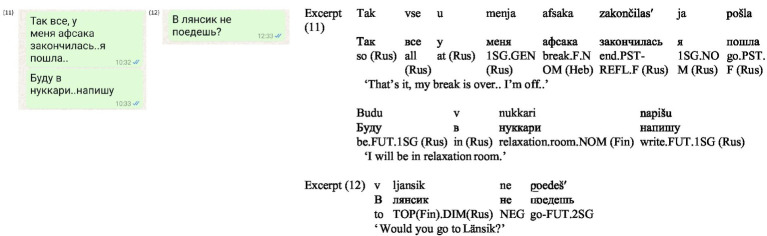
Screenshots of Excerpts (11–12).

The data also reveal cases where translanguaging serves not to describe a place but to index it, associating it with a particular familylect, as illustrated in the following excerpts. In (11) (Figure 7), the parent frames the message in Russian, but uses the Hebrew word for a break—*hafsaka*, followed by the Finnish spoken-language term *nukkari* [relaxation room], typically associated with daycare settings. Upon clarification, the participant explained that it referred to the ‘quiet’ room at work, which had reportedly been discussed numerous times in the family setting. This term further exemplifies its familylectual nature as it was transcribed in Cyrillic script. Such instances highlight how translanguaging mitigates the lack of physical presence and asynchronous interaction in SMS communication. By leveraging language to index spatiolinguistic contexts, participants successfully created a shared sense of ‘being there’ (Spagnolli and Gamberini, 2005).

In (12) (Figure 7), from the same participant, a question that uses a Russian frame and is written in Cyrillic script, applies a topographical indexation *länsik,* showcasing multiple translingual practices. In Finland, two major shopping malls share the name *Länsikeskus*. The participant shortened the name, transcribed it using the Cyrillic alphabet, added the Russian diminutive suffix *-ik*, and did not capitalize the first letter of this proper noun, which likely reflects the participant’s schooling experience, as they studied from the end of primary school through high school in Hebrew, a language that does not use capital letters. The Russian frame ensures comprehensibility, the embedded Finnish place-name indexes shared knowledge, while shortening and the diminutive suffix mark the mall as part of the family’s everyday repertoire.

In addition to indexing places, translanguaging in digital communication is often employed to convey temporal information (Morel and Pekarek Doehler, 2013). This practice allows multilingual individuals to utilize the most precise or contextually appropriate terms from their linguistic repertoire.

**Figure 8:**
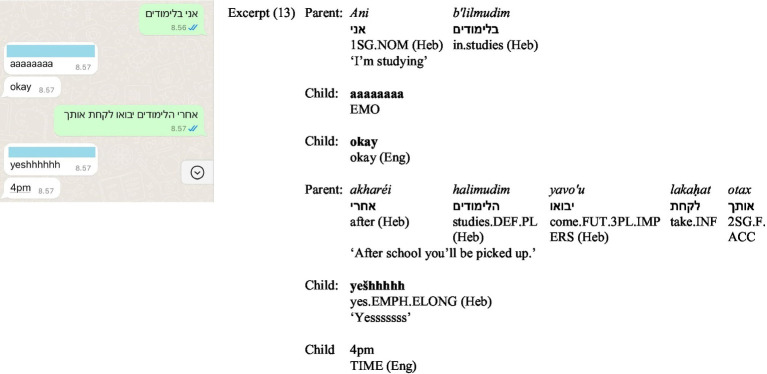
Screenshot of Excerpt (13).

Going back to Excerpt (4), the child combines Russian and Finnish to specify a future timeframe, with *September* written in Finnish, adding clarity and precision within the predominantly Russian sentence. Similarly, in (13) (Figure 8), the exchange between parent and child incorporates Hebrew and English. The parent provides practical information in Hebrew ‘*ani b’lilmodim’* [I’m studying], followed by ‘*akharéi halimudim yavo’u lakaḥat otax’*, [after school you’ll be picked up]. The child responds with an elongated ‘yes’ in Hebrew in Roman script and then specifies the time in English. In this case, Hebrew serves for practical arrangements for the parent, while the child contributes emotional alignment (elongated Hebrew *yes*) and temporal precision (English *4 p.m.*). The use of temporal expressions in a different language serves two simultaneous functions. First, it conveys meaning in a practical and efficient manner: in (4), for a child whose primary language of compulsory education is Finnish, typing *September* in Finnish is most probably faster and more straightforward than toggling to the Russian keyboard or spelling the Russian word in Roman script; in (13), the English *4 p.m.* is significantly shorter and more practical compared to equivalent expressions in the other available languages. Second, such transitions highlight the uniqueness of each communication act, contributing to the formation of familylects through creative translingual practices.

Adopting Spagnolli and Gamberini’s (2005) approach, “place is the set of resources organized and transformed by the involvement in human action at any certain moment” (p. 8). In the examples above, the concepts of place and time extend to the linguistic domain, where language becomes a critical resource for communication and meaning-making. This is evident in how participants adapt and transform translingual practices into familylects within their interactions.

#### 4.2.2 Phatic function

Translingual practices in mobile messaging are consistently linked to a limited range of actions with a strong phatic focus, such as greetings, apologies, and well-wishes (Pekarek Doehler, 2011). In the data, translanguaging was most often utilized for expressing gratitude and encouragement. Examples of greetings were available from one family, where all the family members greeted and bid farewell to each other in Finnish, written in Cyrillic script. Expressions of gratitude appeared in various forms, both language and script; distribution between languages and scripts was equable (Table 1).

**Table 1:** ‘Thanks’: distribution between languages and scripts.

***Script*** / Language	***Roman***	***Hebrew***	***Cyrillic***
Russian	1		3
Hebrew	1	1	1
Finnish	2		2
English	4		

**Figure 9:**
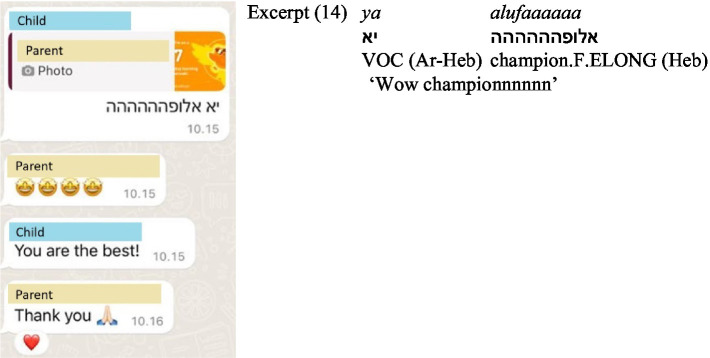
Screenshot of Excerpt (14).

**Figure 10:**
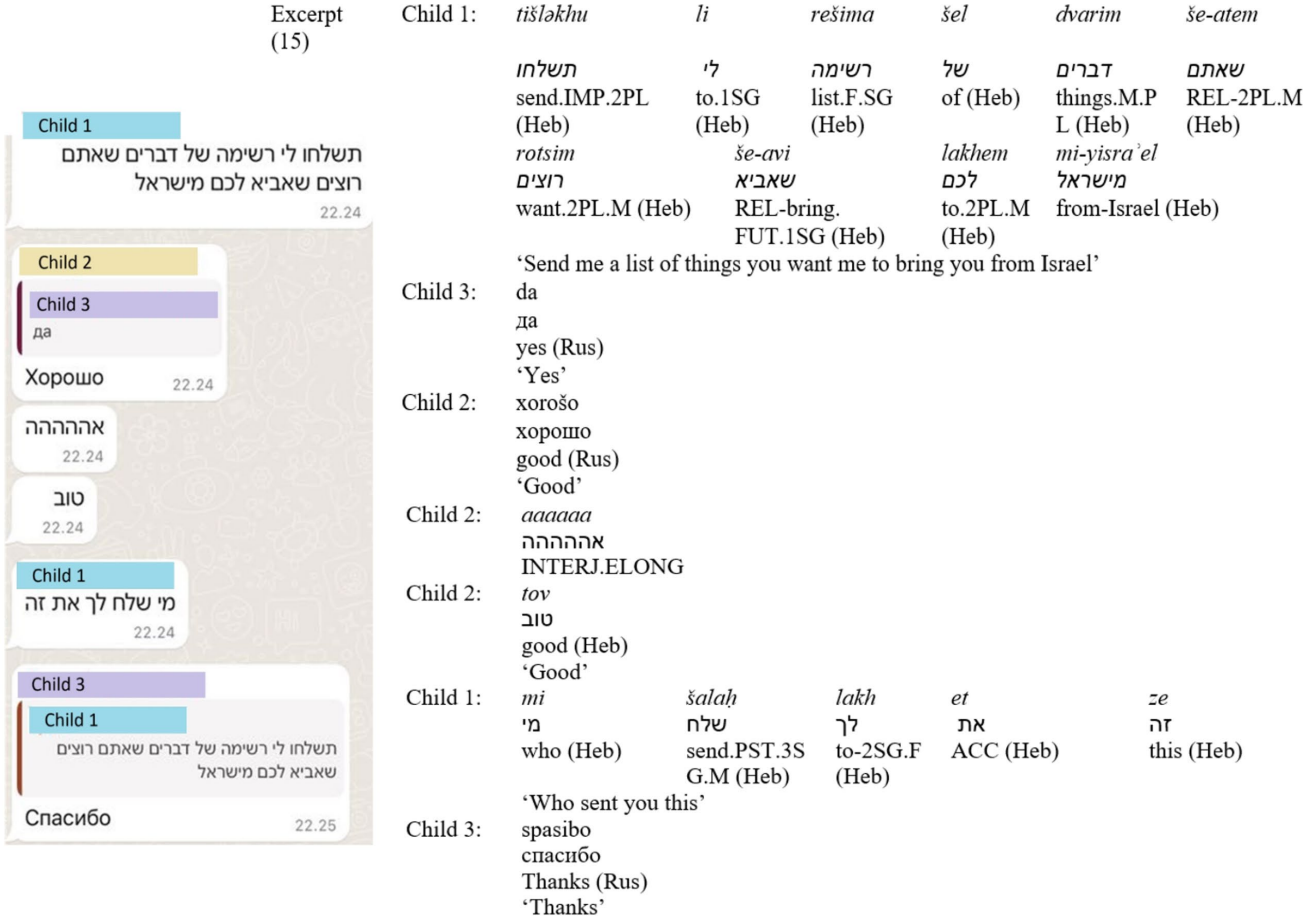
Screenshot of Excerpt (15).

In (14) (Figure 9), the child praises the parent’s Duolingo achievement, first with an Arabic-influenced Hebrew slang expression—*ya* (an Arabic vocative particle used in colloquial Hebrew) combined with *alufa* [champion], and then reinforces it with an English compliment. The parent aligns with the final praise by responding in English. Expressions of agreement and gratitude were frequently repeated by the children in a different language. For instance, in (15) (Figure 10), in a conversation between three siblings, two siblings respond to a statement in Hebrew by the third sibling—‘Send me a list of things you want me to bring you from Israel’ – both in Russian and in Hebrew, using repetition across languages for reinforcement—*xorošo, tov* ([good], Russian, Hebrew).

Another feature observed in (15), as well as twice in both (8) and (13) (see Sections 4.1.3 and 4.2.1), is word stretching, or elongation. Elongated words are common in CMC and relate to sentiment and how they serve as analogues for nonverbal cues such as tone or pitch in spoken language (Gray et al., 2020). They are frequently used to emphasize or exaggerate the meaning of the word (Rafae et al., 2024). In all three cases, the children toggle the keyboards before applying elongation.

**Figure 11:**
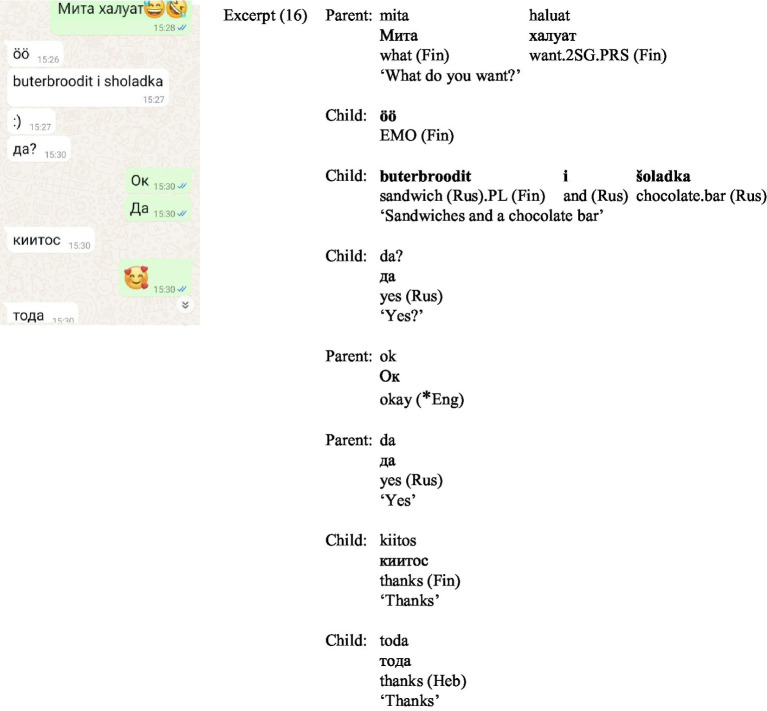
Screenshot of Excerpt (16).

In (16) (Figure 11), the parent begins humorously, posing a question ‘*mita haluat’* [What do you want?] in Finnish transcribed in Cyrillic script about the child’s food choices. The child responds similarly, answering in Russian but using Roman script and adding a Finnish plural suffix ‘-it’ to the Russian word for ‘sandwich’—*buterbrood*. To ensure the parent approves of the (not particularly healthy) food choices, the child follows up with a clarifying question in Russian – *da?* [yes?], written in Cyrillic script. After receiving confirmation from the parent, ‘OK’ and ‘yes’, in Cyrillic script, the child thanks twice, in Finnish (*kiitos*) and in Hebrew (*toda*), both in Cyrillic script. The findings align with Pekarek Doehler (2011), who demonstrated that trans-scripting serves to demarcate phatic actions, playing a key role in enhancing expressivity in translingual SMS communication. Building on these insights, the findings also challenge the assumption that CMC communication prioritizes brevity. Toggling between keyboards can be time-consuming, and even when using a single keyboard, certain expressions, such as thanks written in Russian using Roman script, can be longer than their equivalents in Finnish or English. This emphasis on expressivity and sacrificing brevity is also evident in instances where gratitude is repeated consecutively in two languages, highlighting how multiple languages and script variations serve as tools for reinforcing emotional connections and expressing shared cultural identities within a family.

#### 4.2.3 Address terms

Address terms are a common feature often conveyed through using multiple languages (e.g., Pekarek Doehler, 2011; Istanbullu, 2021; Ndabihonge, 2023). In SMS messages, translingual practices are used for both affectionate and casual address terms, with different languages serving specific purposes. Pekarek Doehler’s (2011) analysis of plurilingual SMS communication in Switzerland found that while English was primarily used for ‘cool’ terms, French was preferred for expressing affection. In contrast, German and Swiss-German were rarely used, suggesting that these languages play distinct roles in SMS communication, particularly in expressing intimacy.

In the data, there were few instances of parents addressing the child by name, and only in Cyrillic script. Two of the six participants were fathers; no specific address terms for them were found in the data. In contrast, addressing mothers was widespread, often initiating communications.

**Figure 12:**
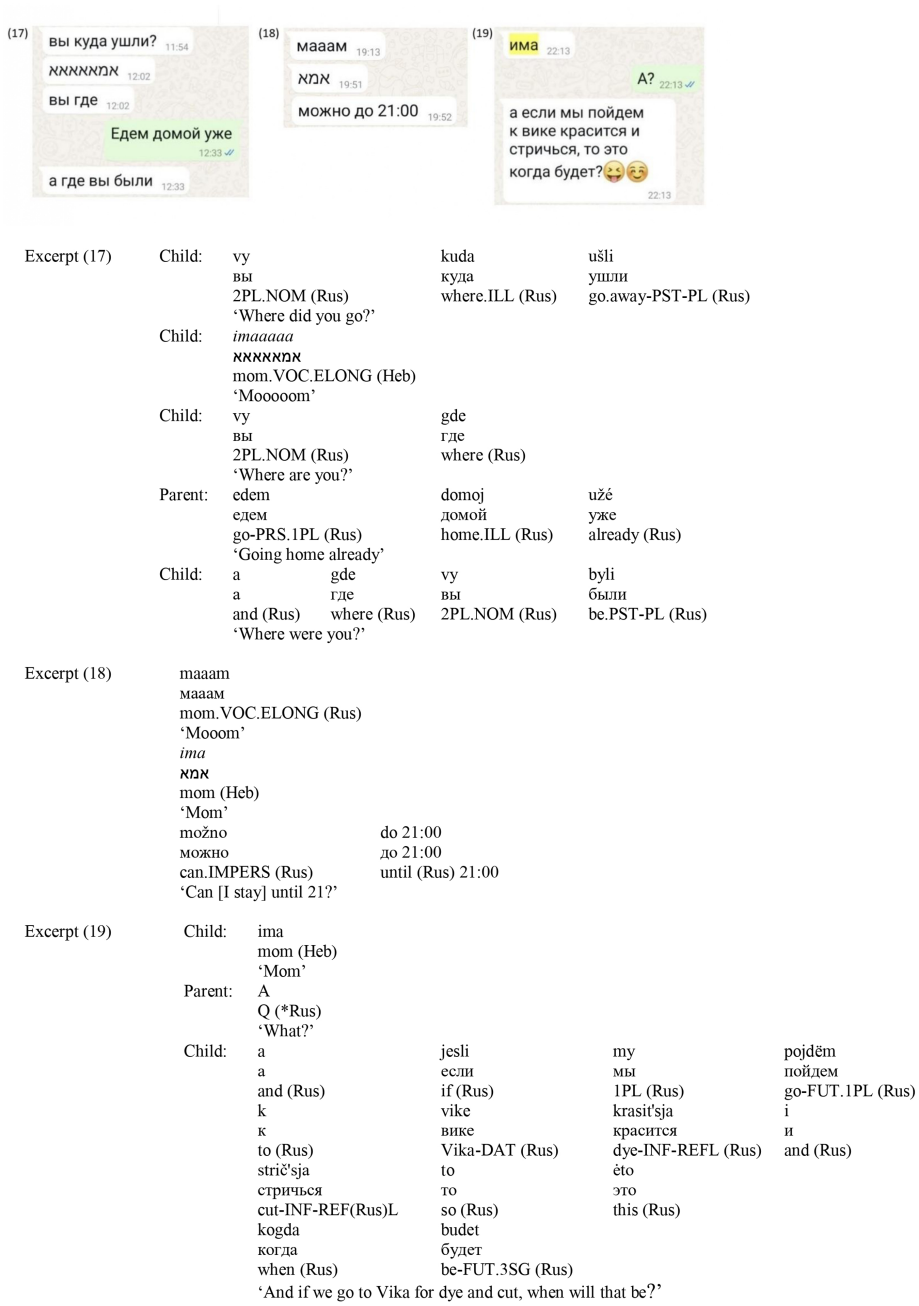
Screenshots of Excerpts (17–19).

In (17) (Figure 12) the conversation is held in Russian, both by the child who asks for the parents’ location, and the parent who responds, but for addressing the mother—*imaaaaa*—the child uses Hebrew and toggles the keyboard. After this, the child returns to Russian. In (18) (Figure 12), the same child first addresses the mother in Russian—*maaam*, then switches to Hebrew – *ima*, then toggles the keyboard and returns to Russian for a request to stay longer. The use of two address terms in quick succession, across languages and scripts, intensifies the appeal and underscores the child’s agency in drawing on all available resources to secure the parent’s attention (cf. Lanza, 2021). The child also uses vowel elongation when addressing the mother with an urgent request, emphasizing the importance of the message (Gray et al., 2020; Rafae et al., 2024). In (19) (Figure 12), the child initiates a request for a beauty treatment with the Hebrew *ima* [mom], followed by a request in Russian, while retaining the Cyrillic keyboard. Taken together, these examples illustrate how address terms for mothers function as affective anchors in multilingual parent–child interaction, with children strategically deploying translanguaging to highlight both intimacy and urgency (Pavlenko, 2004), while also showing children’s active role in achieving interactional goals (cf. Lanza, 2021).

**Figure 13:**
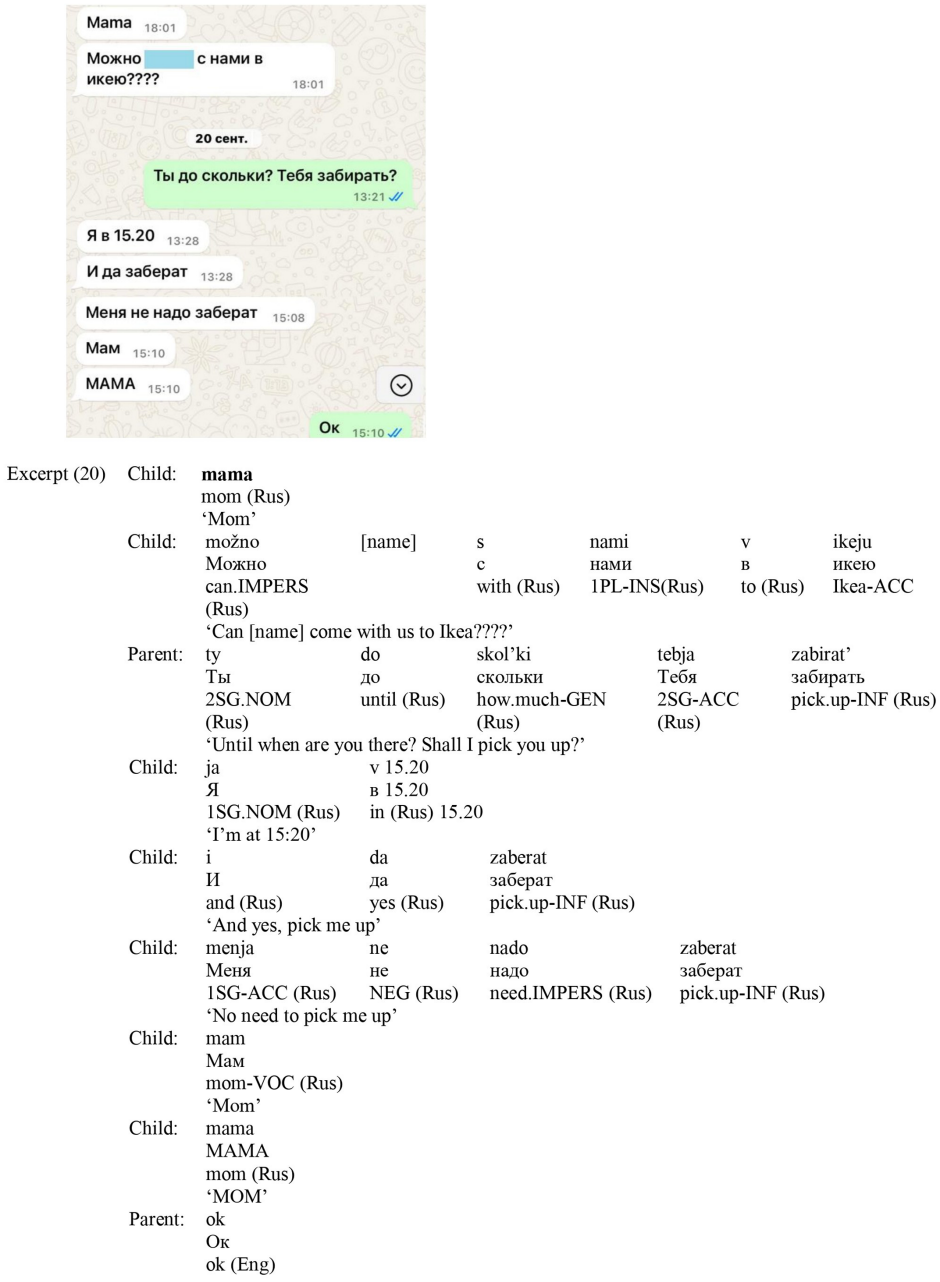
Screenshot of Excerpt (20).

In excerpt (20) (Figure 13), the child addresses the mother in Russian on two consecutive days. On the first day, the child writes *mama* in Roman script and then switches to Cyrillic to ask permission for a friend to join. The following day, the mother replies in Russian, asking when to pick the child up. The child answers in Russian with Cyrillic script, specifying the desired pickup time. A few hours later, the child updates the mother in Russian that pickup is no longer needed, and then repeats the address term twice, first as *Mam* with only the initial letter capitalized, and then as *MAMA* in uppercase, to emphasize urgency. This use of all-caps aligns with the common belief that capital letters are particularly effective for drawing attention and prioritizing important messages, especially when time constraints are involved (Arbel and Toler, 2020).

A broader trend was observed in the data, with Russian being the predominant choice to address mothers, reflecting the emotional significance of Russian for the Gen 1.5 participants, who used only Russian to address their mothers. This linguistic practice appears to have been passed down to their children. Of the 20 instances of addressing mothers in the data, 13 were in Russian, with 9 of them using the Cyrillic script (Table 2).

**Table 2:** ‘Mom’: distribution between languages and scripts.

***Script*** / Language	***Roman***	***Hebrew***	***Cyrillic***
Russian	2	2	9
Hebrew	3	1	2
Finnish	1		

In contrast to the translingual functions discussed in previous sections, address terms in WhatsApp communication reveal significant language preferences, with Russian being the only option of addressing the children by the name (but conclusions are hard to make due to insufficient number of such addresses), and predominating in addressing mothers, most probably due to emotional and cultural factors. This claim aligns with Istanbullu’s (2021) and Pavlenko’s (2004) findings on the role of emotional factors in language choice within multilingual families. Istanbullu’s (2021, 2024) analysis of Arabic- and Turkish-speaking transnational families in Europe shows that Arabic, the language of first socialization of grandparents and parents, was retained across generations in affectionate address terms. This alignment was mutual: grandparents shifted toward national languages—French or German—despite difficulty, while grandchildren aligned toward Arabic, which they themselves found challenging. This mutual adjustment was made possible by the adults’ caring attitude, which encouraged the children to reciprocate. Pavlenko (2004) notes that language choice in emotional expression often reflects deeply ingrained cultural and affective associations. Specifically, she finds that mothers frequently revert to Russian, their first language, for emotionally charged interactions, such as expressing intimacy, affection, or authority, as it is often perceived as more emotionally resonant and authentic. This emotional connection to language is often passed down to the next generation, supporting the idea that the use of Russian by children to address their mothers reflects both emotional ties and cultural continuity. The use of the Cyrillic alphabet to write children’s names, even when those names are not inherently Russian, further highlights the role of language and script as carriers of cultural heritage and emotional expression, transcending the specific linguistic origins of the names themselves.

## 5 Discussion

This study explores translanguaging practices in WhatsApp text messaging in Israeli families with a PSS background residing in Finland. Through the micro-interactional lens, the findings highlight how family members negotiate their multilingual resources to achieve various communicative goals. The primary objective of this study was to investigate the translingual messaging practices within a family setting, as reported by the six participants, and the functions linked with these choices. Given the highly dynamic nature of language use and the evolving contexts in which transnational multilingual families operate, there is an increasing need to continuously investigate their language policies and practices (Hirsch and Lee, 2018; Lanza, 2021).

The findings show that translanguaging is the central means through which transnational multilingual families shape and negotiate their everyday language practices in text messaging. The use of translanguaging in WhatsApp communication in these families serves various functional purposes, especially in parent–child interactions. This study explores three main functions of translingual practices identified in the data: local and temporal functions, phatic functions, and address terms, emphasizing how these practices address both practical communication needs and emotional expression. In parent–child CMC, the two primary reasons for using resources from the variety of languages available revolve around managerial and emotional communication (e.g., Fletcher et al., 2018; Janssen et al., 2024). Interpreted through this framework, the present study data show that the local and temporal functions mainly address managerial needs, conveying practical details like time and place. In contrast, phatic functions and address terms are more associated with emotional communication, expressing affection, identity, and familial connection. That is, using specific languages to include references to particular time, such as S*eptember* (Excerpt 4) and *4 p.m.* (Excerpt 13), helps convey practical details with more precision, while toggling keyboards and / or switching the language for expressing encouragement or gratitude (Excerpts 14–16) align with emotional communication. Word elongation (Excerpts 8, 13, 15) and uppercase (Excerpt 20) can amplify emotional significance to express urgency or significance of the address. These instances reflect the broader function of pragmatic borrowings, which are typically used to express emotions, attitudes, evaluations, and interactional stances rather than factual or propositional content (Peterson, 2017; Verschik and Kask, 2023).

This similarity in functions highlights how translanguaging practices serve both practical communication needs, like managing time or place, and emotional needs, such as expressing affection or identity, though their frequency and importance can vary across different contexts. The present study revealed strategic functions of translanguaging practices similar to those identified by Ndabihonge (2023) in multilingual WhatsApp communication in French, Kirundi, Kiswahili, and English—economy of communication, identity signaling, expressivity, and filling lexical gaps—though with a different order of preferences. While the economy of communication was the most recurring theme in the Burundian context, in the current study, the use of shorter, more practical terms was often secondary to other functions. Numerous instances of translanguaging and trans-scripting for addressing mothers and expressing gratitude prominently illustrate how expressivity and identity signaling can take precedence over the economy of communication. In several cases, children switched keyboards, often toggling back afterward, specifically to address their mothers in a language they believed would be more emotionally meaningful (e.g., Excerpts 17–19).

The function of translanguaging is particularly important in families that maintain multiple languages in their repertoire, as it allows speakers to draw definitively on diverse linguistic resources in order to sustain intergenerational communication and relational closeness (Palviainen and Räisä, 2023; Istanbullu, 2024). In the present study, participants used translanguaging to express concepts or ideas with terms that were perceived as most suitable for capturing the intended meaning. The examples include terminology connected to school, including *high school* (Excerpt 8) and *domestic science* (Excerpt 9), specific places like *relaxation room* (Excerpt 10), and food names, like *stir-fry* (Excerpt 7). This reflects a pragmatic choice aimed at minimizing linguistic effort, making communication more efficient and precise.

Another key feature of the translanguaging practices observed in this study was identity signaling. Language choice in a multilingual conversation is a powerful marker of identity (e.g., Gross et al., 2022). In the data, Russian was the most frequently used language by the children to address their mothers, reflecting emotional closeness and cultural heritage. This practice emphasizes Russian as a familial language, reinforcing a deep emotional connection, especially since in the participants’ families it is also the predominant language spoken with the grandparents (Bloch, 2024b). Schwartz and Verschik (2013) argue that the intergenerational transmission of a heritage language is deeply emotional, as it evokes memories of the parents’ own childhoods. Switching to Russian when addressing the mothers signals that this language functions not only as a means of communication but as a shared emotional resource. Children draw on inherited emotional associations by intuitively selecting Russian in moments of emotional significance, not for efficiency, but for reinforcing intimacy and shared family identity. These findings correspond with Istanbullu’s (2021, 2024) analysis of Arabic- and Turkish-speaking transnational families in Europe, where affectionate address terms in Arabic exchanged across generations supported the maintenance of heritage Arabic, with reciprocal language choices and agency serving as key factors in intergenerational communication. In this study, the children’s recurrent use of Russian when addressing their mothers illustrates a reciprocal alignment through which Russian’s emotional and familial role is evident.

The data also highlight the creative role children play in shaping the translingual practices within the family. Frequent keyboard toggling, word elongation, and use of school-related terms reflect child agency in FLP (Lanza, 2021). While parents set the initial framework for language use based on their own language ideologies and management strategies, their children reshape it through WhatsApp interactions. This finding supports Nenonen’s (2024) claim that translanguaging is a co-constructed process driven by children’s own linguistic environments and social identities, not merely a parental strategy.

## 6 Conclusion

This study explores translanguaging practices in the WhatsApp communication of transnational Russian-Hebrew-speaking families in Finland. The findings demonstrate that language choices are strategically employed to meet both practical and emotional communication needs. The three primary functions observed in the data—local and temporal, phatic, and address terms—serve to enhance the clarity of practical information and strengthen familial bonds. The strategic blending of Russian, Hebrew, Finnish, and English reflects not only a response to immediate communication needs but also allow for the signaling of identity and emotional connection within the family unit. The familylects formed through these practices reflect the dynamic integration of various languages, contributing to the formation of family-specific linguistic repertoires that serve as important markers of identity and connection, as well as address practical communication needs. Another key aspect of this study findings is the unique integration of practical and emotional functions within translingual practices, tailored to the specific linguistic and cultural contexts of these families. These insights extend beyond the family unit, offering a broader understanding of how multilingual families handle complex social and linguistic environments. The findings also highlight the role of digital communication in preserving cultural heritage and fostering linguistic adaptability. Future research could adopt a longitudinal approach to capture how language practices in transnational multilingual families evolve over time, as well as explore different language combinations to examine how family dynamics unfold across diverse multilingual contexts.

## Data Availability

The original contributions presented in the study are included in the article/supplementary material, further inquiries can be directed to the corresponding author.

## Footnotes

^1^While the term *language of first socialization* (Istanbullu, 2024) is commonly used in the sociolinguistics of multilingualism, particularly in analyzing language practices of multilingual families and individuals, this study adopts the broader concept of *heritage language* - an ethnolinguistic minority language acquired in contexts of early bilingualism or multilingualism, typically within the family (Montrul and Polinsky, 2021), or even “a language of personal relevance” distinct from the majority language (Van Deusen-Scholl, 2003, p. 216). This broader definition is necessary because it cannot be confirmed whether Russian was the actual first language of socialization for the participants. Born in different Soviet republics, in early daycare settings they may have first encountered either Russian, the USSR’s dominant language (Dietrich, 2005), or the language of the republic (e.g., Ukrainian or Kazakh). Regarding home environments, one participant’s parent was neither a Soviet citizen nor a Russian-speaker, having immigrated to the USSR through marriage, further complicating assumptions about Russian as the language of first socialization.
